# Identifying community healthcare supports for the elderly and the factors affecting their aging care model preference: evidence from three districts of Beijing

**DOI:** 10.1186/s12913-016-1863-y

**Published:** 2016-11-15

**Authors:** Tianyang Liu, Xiaoning Hao, Zhenzhong Zhang

**Affiliations:** China National Health and Development Research Center, Beijing, 100191 China

## Abstract

**Background:**

The Chinese tradition of filial piety, which prioritized family-based care for the elderly, is transitioning and elders can no longer necessarily rely on their children. The purpose of this study was to identify community support for the elderly, and analyze the factors that affect which model of old-age care elderly people dwelling in communities prefer.

**Methods:**

We used the database “Health and Social Support of Elderly Population in Community”. Questionnaires were issued in 2013, covering 3 districts in Beijing. A group of 1036 people over 60 years in age were included in the study. The respondents’ profile variables were organized in Andersen’s Model and community healthcare resource factors were added. A multinomial logistic model was applied to analyze the factors associated with the desired aging care models.

**Results:**

Cohabiting with children and relying on care from family was still the primary desired aging care model for seniors (78 %), followed by living in institutions (14.8 %) and living at home independently while relying on community resources (7.2 %).

The regression result indicated that predisposing, enabling and community factors were significantly associated with the aging care model preference. Specifically, compared with those who preferred to cohabit with children, those having higher education, fewer available family and friend helpers, and shorter distance to healthcare center were more likely to prefer to live independently and rely on community support. And compared with choosing to live in institutions, those having fewer available family and friend helpers and those living alone were more likely to prefer to live independently and rely on community. Need factors (health and disability condition) were not significantly associated with desired aging care models, indicating that desired aging care models were passive choices resulted from the balancing of family and social caring resources.

**Conclusions:**

In Beijing, China, aging care arrangement preference is the result of balancing family care resources, economic and social status, and the accessibility of community resources. Community facilities and services supporting elderly were found to be insufficient. For China’s future health system, efforts should be made to improve community capacity to provide integrated services to senior citizens.

**Electronic supplementary material:**

The online version of this article (doi:10.1186/s12913-016-1863-y) contains supplementary material, which is available to authorized users.

## Background

“In serving his parents, a filial son reveres them in daily life; he makes them happy while he nourishes them; he takes anxious care of them in sickness; he shows great sorrow over their death that was for him; and he sacrifices to them with solemnity.”— Confucius [1].

Filial piety is an age-old corner stone of Chinese culture. Intergenerational cohabitation features as a customary household structure [2]. Demographic and economic changes in the last three decades include smaller families and more affluence. Chinese family structures have transformed from extended to nuclear. Intergenerational cohabitation rates are fading, especially in urban areas [3, 4]. As the first generation of the only children is entering their thirties and forties, they become the so called “sandwich generation” who need to support both their children and parents. Many seniors who don't want to burden their adult children choose to live independently or in institutions [5]. By the end of 2014, China had more than 212 million elderly or people older than 60 years, accounting for around 15.5 % of the country’s total population [6]. As the traditional support system is in a time of transition, the elderly can no longer rely on their children as was previously the case. Who then will care for the elderly? What choices do they have for living the last stages of life?

The care resources for elderly mainly come from three sources: family, institutions and the community. The elderly rely on their children in a number of ways. Traditionally, they live with their oldest male child. Sometimes widowed parents live with their children’s family by turns [7]. Nowadays most of the elderly still prefer to live at home, with surveys showing preferences ranging from 54.9 to 96.7 % [5, 8–11]. While in urban areas, when financially possible, “living closely but separately” is more popular. Shuqin Long found that although 95 % of urban elderly people would like to live at home, 74 % of them prefer living at home independently for the reasons including greater freedom, less burden to children, limited room, and poor relationships with their children [5].

By 2010, there were 34,100 multi-functional aging care institutions in China. These institutions had 5.5 million beds with an occupancy rates around 55 % [12]. Most of them provided daily life assistance, but not sufficient medical care resources – according the China National Research Center on Aging, less than 60 % of the aging care institutions had an infirmary [13]. Gu’s research indicated that elders who were younger, male, residing in urban areas, with lower family-care resources, and exhibiting poorer health were more likely to live in institutions rather than in communities [14].

Currently available community resources serving the senior citizens are divided into in-system and out-system resources [15]. In-system resources mainly include city neighborhood committees and community health centers (CHCs). Out-system resources include private service providers (e.g. housekeeping companies, private day-care centers, home-based care centers, etc.) and community facilities (e.g. elderly activity centers, body-building gyms, chess and cards rooms, green areas, etc.). The government requires that one neighbourhood committee should be set up for every community with 1000 to 3000 households. At the end of 2014, there were 96,693 urban neighbourhood communities and 585,451 village neighbourhood communities in China [16]. The CHC is the major healthcare resource for community residents. CHC functions include prevention, health education, women and children’s care, elderly care, immunization, and physical rehabilitation [17]. The CHC is responsible for making health profiles, managing people’s chronic diseases (e.g. recording their blood sugar and blood pressure four times a year), and conducting free physical examinations for the elderly residents living in the community. At the end of 2014, there were 34,238 CHCs with 195,900 beds, with an occupancy rate of 55.6 % and 685 million annual visits [16].

Based on these elderly care resources, there are now three common aging care models for China’s elderly: cohabiting with children and relying on the care from family – the traditional way; living at home independently and relying on the care from community; and living in an institution [5]. Many elderly people in western countries prefer to live in their own homes and live independently, and policies have favored the move from institutionalized care to community-based care [18]. In China, the Law on the Protection of the Rights and Interests of the Elderly also mentions that “The state shall establish and improve the social elderly care service system which is based on families and supported by communities and institutions.” [19]. Lu Xinping also proposed that although institutionalized care had its historical and practical origins in China, community based care for the elderly was more attractive in terms of long-term economic and humanitarian perspectives [20].

The community capacity of proving social and healthcare service is an important factor to consider when choosing care models for the elderly. Based on the support from family members and outside, living at home (cohabiting with children or living at home independently) still remained the priority of most elderly people [5, 8–11]. However, although many studies analyzed the associated factors affecting the choices of care models of urban elderly [5, 10, 11, 20, 21], few of them examined the effect of community capacity except for the study conducted by Zhang Wenjuan [10], highlighting the failure to recognize the possibility of enriching the choices of elderly by improving community capacity.

There are several studies that have examined the determinants of aging care preference in China. Three studies [5, 21, 22] used cohabitation with children or not as the dependent variable; another two studies used living in institutions or not [10, 23]. To date, no studies in China have examined more than two categories of aging care models. In this study, we identify the community support for the elderly, and analyze the associated factors affecting the choice among three desired aging care models of the elderly dwelling in communities: cohabiting with children, living independently, and living in institutions. We apply the Andersen-Newman Behavioural Model, explain in detail below, which provides a framework for viewing health services utilization [24] and is used by many Chinese studies to explore factors that lead to personal health choices and healthcare services utilization patterns among the elderly [2, 10, 25, 26]. This study takes community factors into consideration, to explore how to improve community capacities to better service the elderly. More specifically, the following questions are addressed:

- What facilities and services supporting the elderly exist in Beijing?
- What are the elderly’ expectations for the community capacity?
- What are the factors affecting the elderly’s choices for future aging care?
- Do community capacity factors affect willingness to choose various aging care models?

## Methods

### Data and sample

**Fig. 1:**
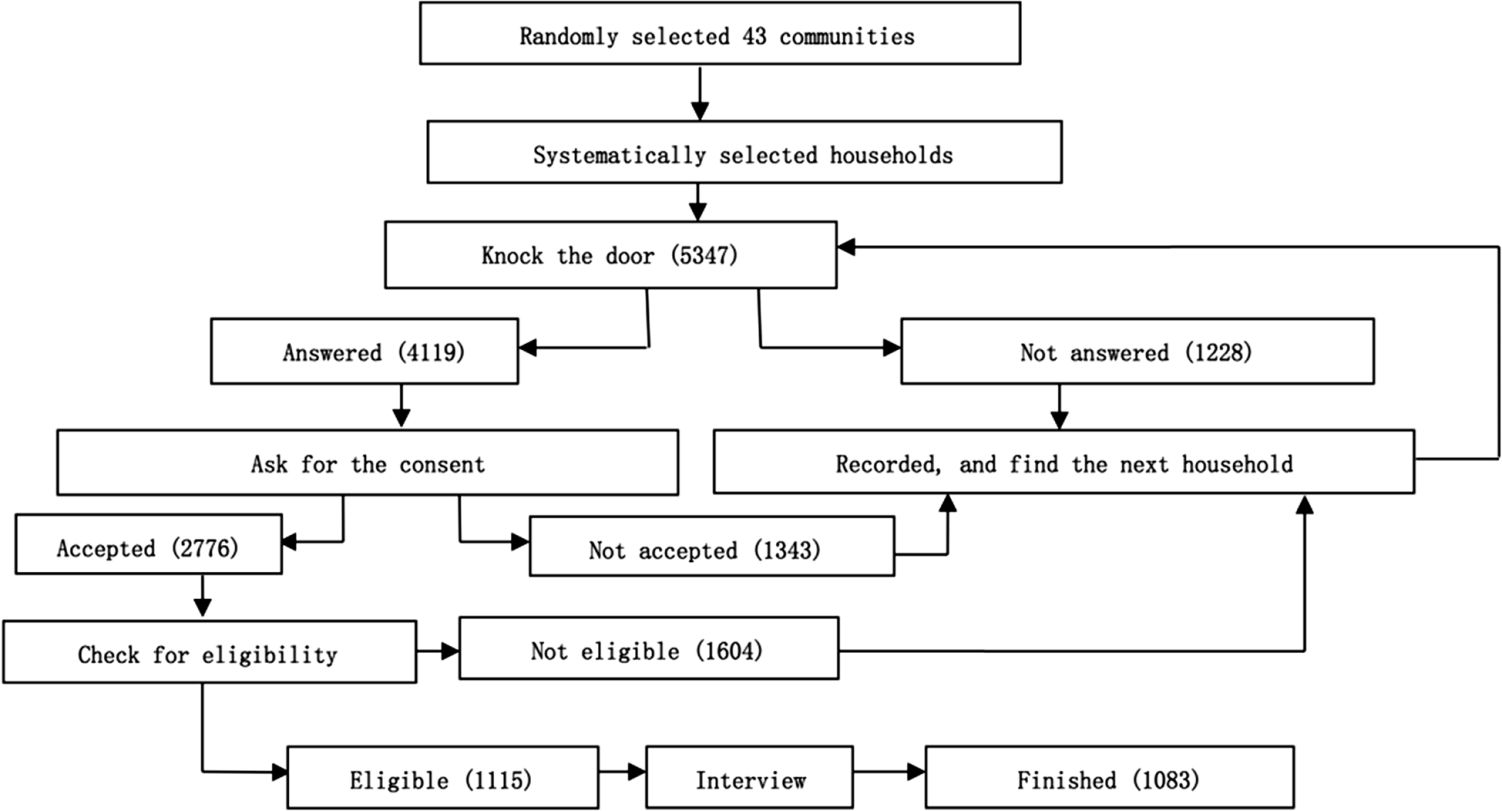
Sampling flow of elderly living in household within communities

This study used the survey “Health and Social Support of Elderly Population in Community”. The questionnaires were issued between January and May 2013, covering three different functional districts (Xicheng district of political and culture function, Chaoyang district of extended urban function and Tongzhou district of new development function) of Beijing, which included 43 communities (villages). Initial sample size (1067) was calculated by the formula of *N* = Z^2^× (P × (1-P))/E^2^ (Z = 1.96, E = 3 %, and *P* = 0.5), and then distributed by district, age and gender (according to the district, age and gender distribution of all the elderly citizens in the three districts, see Additional file 1) [27]. China National Health Development Research Center Review Board reviewed, approved and implemented the study. Twenty trained interviewers went to the communities that were randomly selected in each district and systematically visited households in the community to find eligible interviewees. Elders aged more than sixty, living in the community (village) for more than 6 months, having Beijing citizenship, and having the mental capability for the interview were eligible to participate in the face-to-face structured questionnaire interview. Finally 1083 elders were successfully interviewed, see Fig. 1.

### Dependent variable

The interviewees were asked the question “What’s your most desired aging care model in the future?” They could choose from three given options: (1) Cohabiting with children, and relying on care provided by children or spouse; (2) Living independently at home, relying on community aging care resources, for example day care center, home based health and life assistance services; (3) Living at an institution, for example nursing home and elderly apartment. If they had no aging care model preference for future, they could skip the question.

### Independent variables

As mentioned above, the Andersen-Newman Behavioural Model was adopted as the conceptual framework. This framework traditionally consisted of three domains as individual determinants of utilization: predisposing factors, enabling factors, and need factors. Predisposing factors could be characteristics such as demographic, social structure and health beliefs. Enabling factors were those which unable or impede the use of health service, such as family support and access to health insurance. Need factors represented both perceived and actual need for health care services. This study included variables that were commonly used in Andersen domains, and added four available community variables as anther domain to examine their effect on elders’ preference of aging care arrangements.

Predisposing characteristics represented elder’s predisposition in the preference of future aging care arrangements. Age, gender, and education level factors were included, which indicated biological and social imperatives suggesting the likelihood of elders’ preference. Enabling variables were those enabling or impeding the preference of future aging care models. Monthly income was already proved to be a significant predictor of aging care model preference [10, 21] and the utilization of healthcare services among elderly [25]. Current living situation (live alone; with spouse only; cohabiting with children or others) was also crucial because people often anchored from what was to what was best. They preferred what they had. Number of family and friend helpers (sum of spouse, children and closely contacted friends that could help the elders with personal care) represented the size of available informal caregivers, as well as the social support for the elderly. This variable was developed by Bass’s study in exploring the effect of family caregivers on elder’s use of in-home services [28].

Need variables were represented by the actual need of care and health services, including chronic disease, ADL and IADL. The survey accessed the functional disability of the elderly through simplified Lawton and Brody’s Activity of Daily Living Scale (ADL) and Instrumental Activities of Daily Living Scale (IADL) to measure elderly functional impairments [29]. ADLs composed of six items including feeding, bathing, dressing, toileting, grooming and physical ambulation. IADLs included seven items considered to have ADL or IADL impairments. For ADLs, the total score ranges from 0 to 6, and for IADLs, from 0 to 8.

Community variables were added as a new domain of the Andersen’s Model. Elders were asked questions about their knowledge and perception of the facilities and services existed in the community: does your community have an elderly service center providing home-based care?; does your community have an activity center for senior citizens?; does the community have day-care center for senior citizens?; what’s the distance from your home to the nearest community healthcare center? These community factors represented what services were available and perceived by elderly within the community. See Table 1.

**Table 1:** Descriptive statistics

				Preferred aging care arrangements (%)			Likelihood ratio test (*p*-value)
			Total	Cohabiting with children, cared by family	Living at home independently, cared by community	Institutions	
				*N* = 809	*N* = 74	*N* = 153	
Total				78 %	7.2 %	14.8 %	
Predisposing variables	Age	60–69	43.4 %	75.6 %	7.3 %	17.1 %	0.492
		70–79	39.9 %	80.6 %	6.8 %	12.6 %	
		80+	16.7 %	78.6 %	7.5 %	13.9 %	
	Gender	Male	49.6 %	78.0 %	7.2 %	14.8 %	0.954
		Female	50.4 %	78.2 %	7.1 %	14.8 %	
	Education	Illiterate	11.8 %	81.1 %	8.2 %	10.7 %	0.000
		Primary-	30.5 %	83.9 %	5.7 %	10.4 %	
		Secondary	48.6 %	77.3 %	5.8 %	16.9 %	
		Tertiary+	9.2 %	58.9 %	17.9 %	23.2 %	
Enabling variables	Monthly income	Less than 3000	33.2 %	85.5 %	4.4 %	10.2 %	0.019
		3000+	66.8 %	74.4 %	8.5 %	17.1 %	
	Current live situation	Alone	8.1 %	59.5 %	20.2 %	20.2 %	0.002
		With spouse only	40.6 %	78.1 %	6.7 %	15.2 %	
		Cohabiting with children or others	51.3 %	81.0 %	5.5 %	13.6 %	
	Number of family-friend helpers	0–3	9.3 %	8.7 %	17.6 %	8.5 %	0.05
		3+	90.7 %	91.3 %	82.4 %	91.5 %	
Need variables	Chronic disease	0	76.6 %	76.6 %	7.7 %	15.7 %	0.029
		1+	23.4 %	83.1 %	5.4 %	11.6 %	
	ADL	0	78.9 %	77.8 %	6.7 %	15.4 %	0.465
		1+	21.1 %	79.0 %	8.7 %	12.3 %	
	IADL	0	68.6 %	77.2 %	6.9 %	15.9 %	0.989
		1+	31.4 %	80.0 %	7.7 %	12.3 %	
Community variables	Home Based Care center	Yes	6.3 %	80.0 %	3.1 %	16.9 %	0.328
		No	93.7 %	78.0 %	7.4 %	14.6 %	
	Day-care center	Yes	1.5 %	81.3 %	12.5 %	6.3 %	0.207
		No	98.5 %	78.0 %	7.1 %	14.9 %	
	Activity center	Yes	35.0 %	77.7 %	6.6 %	15.7 %	0.516
		No	65.0 %	78.3 %	7.4 %	14.3 %	
	Distance to healthcare center	1 km or less	64.9 %	73.4 %	8.5 %	18.2 %	0.000
		2 km or more	35.1 %	86.8 %	4.7 %	8.5 %	

### Data analysis

Descriptive analyses of the data were carried out by using the SPSS 16. As aging care preference was a three-category dependent variable, multinomial logistic regressions were performed to examine the contribution of predisposing, enabling, need and community factors.

## Results

### Description of the sample

One thousand thirty-six of the 1083 individuals were included in the sample, after excluding 47 elderly who did not report their future aging care preference.

Characteristics of the sample were shown in Table 1. The sample averaged 71.4 years old (standard deviation of 8.16), and approximately half were male (49.6 %). The majority of respondents had secondary education (48.6 %), had more than 3 available family members and friends who could support them with informal care (90.7 %), and a monthly income more than 3000 Yuan (66.8 %) (approximately US $458). About half (51.3 %) of the respondents were cohabiting with children or others, 40.6 % of them were living with spouse, and only 8.1 % of them were living alone. As for health status, about 23.4 % of them had at least one chronic disease, 21.1 % had at least one ADL disability and 31.4 % had at least one IADL disability.

Among the 1036 seniors, the majority (78 %) of them would like to cohabit with children and be cared for by family members for their remaining years. Another 153 (14.8 %) seniors would like to live in institutions, and only 74 (7.1 %) wanted to live at home independently and rely on community caring resources. This indicated that the traditional aging care model of living at home and being cared for by family members was still preferred by most of the elderly, followed by living in institutions and relying on communities. The finding also replicated Tao’s study conducted in Xicheng District, Beijing, which showed that cohabiting with children and relying on the care from family was the primary desired aging care model for seniors (71 %), followed by living at home independently while relying on social service (14.8 %) and living in institutions (14.3 %) [30].

Likelihood ratio test showed that education, monthly income, current living situation, number of family and friend helpers, chronic disease and distance to CHCs had a significant contribution to the aging care model preference (see Table 1).

### Community resources and support for the elderly

When asking the elderly about their knowledge on the facilities and services that could support them within their communities, the results showed that 35 % of the elderly had activity centers in their communities, and only 6.3 % of them had centers providing home-based care in the community, while nearly all of them (98.5 %) did not have a day-care center in their community. More than half (64.9 %) of the elderly have community healthcare centers within one kilometre of their residence, and nearly 89 % of them one within two kilometers. See Table 2.

**Table 2:** Elderly-supportive community facilities, based on respondent reports

Facilities existed in the community that support the elderly	Have	Don’t have
Services		
Home-based daily life assistance	2.1 %	97.9 %
Home-base healthcare, and health knowledge delivery	5.7 %	94.2 %
Shopping assistance	1.8 %	98.1 %
Legal aid	15.8 %	84.2 %
Facilities		
Home-based care center	6.3 %	93.7 %
Elderly activity center	35 %	65 %
Day-care center	1.5 %	98.5 %
Distance to the nearest healthcare center		
Less than 1 km	64.9 %	
1–2 km	24.2 %	
2–3 km	4.8 %	
3–4 km	4.0 %	
4–5 km	2.0 %	
More than 5 km	0.1 %	
Elderly expectations for the community facilities	Need	Not need
Elderly activity center	82.30 %	17.60 %
Home-based care center	85.4 %	14.6 %
Day-care center	83.5 %	16.5 %
Elderly library	75.80 %	24.30 %
Healthcare center	94.60 %	5.40 %
Accessibility facilities (like ramps and roadside armrest)	90.20 %	9.90 %
Green area (planting)	92.80 %	7.20 %
Outdoor activity center	90.70 %	9.30 %
Body-building apparatus	84.80 %	15.10 %
Public benches	95.80 %	4.20 %

As for the services elderly could receive from the community, only 5.7 % of them had access to home-based healthcare services, and nearly none had access to home-based daily life assistance or shopping assistance. About 15.8 % reported that they could receive legal aid assistance from the community commission.

When asking the seniors’ expectations for the community, the top five facilities or equipment that the elderly respondents wanted were public benches (95.8 %), healthcare centers (94.6 %), green areas (92.8 %), outdoor activity centers (90.7 %), and accessibility facilities (90.2 %). There was lower interest in elderly libraries (75.8 %), and this may relate to the low education level of the respondents, among which only 9.2 % had tertiary education.

### Factors associated with elderly future aging care arrangements preferences

The multinomial logistic regression model was presented in Table 3. Wanting to live independently at home and rely on community resources served as the reference category, which was compared with wanting to cohabit with children and live in institutions, respectively.

**Table 3:** Multinomial logistic regression

			Preferred aging care model^a^			
			Cohabiting with children, cared by family		Professional institutions	
			OR (95 % CI)		OR (95 % CI)	
Predisposing variables	Age	60–69	0.556	(0.222–1.394)	0.641	(0.226–1.821)
		70–79	0.858	(0.388–1.898)	0.676	(0.270–1.964)
		80+ (Ref.)			.	.
	Gender	Male	1.026	(0.614–1.714)	0.959	(0.535–1.719)
		Female (Ref.)	.		.	.
	Education	Illiterate	2.704	(0.948–7.717)	0.917	(0.265–3.175)
		Primary-	4.399***	(1.940–9.975)	1.553	(0.603–4.000)
		Secondary	4.199***	(2.067–8.531)	2.405*	(1.081–5.353)
		Tertiary + (Ref.)	.	.	.	.
Enabling variables	Monthly income	Less than 3000	2.225*	(1.165–4.249)	1.498	(0.715–3.318)
		3000+ (Ref.)	.	.	.	.
	Live situation	Alone	0.195***	(0.096–0.396)	0.444*	(0.194–0.905)
		With spouse only	0.920	(0.510–1.685)	0.882	(0.454–1.714)
		Cohabiting with children or others (Ref.)		.	.	.
	Number of family-friend helpers	0–3	0.409*	(0.202–0.828)	0.411*	(0.174–0.969)
		3 + (Ref.)	.	.	.	.
Need variables	Chronic disease	0	0.652	(0.333–1.274)	1.137	(0.526–2.456)
		1+ (Ref.)	.	.	.	.
	ADL	0	1.771	(0.672–4.664)	1.835	(0.595–5.653)
		1+ (Ref.)	.	.	.	.
	IADL	0	0.982	(0.384–2.514)	0.938	(0.319–2.758)
		1+ (Ref.)	.	.	.	.
Community variables	Home Based Care center	Yes	2.566	(0.524–12.556)	3.144	(0.582–16.988)
		No (Ref.)	.	.	.	.
	Day-care center	Yes	0.360	(0.068–1.907)	0.105	(0.008–1.317)
		No (Ref.)	.	.	.	.
	Activity center	Yes	1.330	(0.769–2.299)	1.256	(0.676–2.335)
		No (Ref.)	.	.	.	.
	Distance to healthcare center	1 km or less	0.489*	(0.272–0.879)	1.183	(0.594–2.354)
		2 km or more (Ref.)	.	.	.	.

*CI* confidence interval

*p < 0.05, ***p < 0.001

^a^The reference category is living independently at home is the reference group. Nagelkerke’s pseudo-*R* ^2^ = 0.14

For predisposing factors, education was a significant factor determining desired living arrangement choices among the elderly. We found that having received tertiary or higher education increased the probability of wanting to rely on community resources rather than on family care and living in institutions. Previous studies indicated that those having higher education were less likely to cohabit with children [5, 22, 23, 31]. One explanation was that lower education was usually accompanied with lower income and as well as a stronger traditional sense of filial piety.

For enabling factors, those with monthly income less than 3000 Yuan were 2.2 times more likely to want to rely on their family’s care compared with relying on community resources (OR: 2.225, 95 % CI: 1.165–4.249). Economic status reflects the elderly’s purchasing power for community or institution services. Many studies discovered that economic status limited people’s choices of aging care model, but in different ways. Our result replicated the findings of most previous studies in China that showed poorer elders had lower intention to be institutionalized [5, 14, 32], although a study by Yabo [23] discovered that poorer elderly had a higher probability of living in institutions. One explanation for the difference was that Yabo’s sample included more “three no” elders (who had no living children, little or no income, and no physical ability to work) that could receive free entry and services from institutionalization. Despite the different directions of the effect, higher economic resources meant more freedom in choosing aging care models and higher expectations for the quality of aging care service provided by community or institutions.

Social supports were crucial in providing care at home. The availability of informal care from family and other sources, such as friends or neighbours were the primary care resources for the elderly [10, 22, 33, 34]. In our study, social support (number of available family and friend helpers) and current living arrangements all affected the desired aging care model of elderly significantly. The effect of living alone was positive on the probability of choosing to rely on community resources. While elders having fewer than 3 family and friend helpers were more likely to rely on communities compared with living at home (OR:0.409, 95 % CI:0.202–0.828) and living in institutions (OR:0.411, 95 % CI:0.174–0.969).

Need variables including whether having chronic disease, ADL and IADL disabilities were not significant factors determining aging care model preference. As for community variables, the result showed that people with community health care centers in less than 1 km from their home were more likely to rely on community resources compared with family care (OR: 0.489, 95 % CI:0.272–0.879).

## Discussion

This study focused on the effect of community capacity in choosing aging care models. The results suggested that community facilities and services supporting the elderly were underdeveloped in Beijing. In the sample districts, although more than half (64.9 %) of the elderly have community healthcare centers in less than one kilometre, access to other community services and facilities (home-based assistance and healthcare, legal aid, home-based care center, elderly activity center and day-care center, etc.) remained very low, despite the fact that these resources were needed and expected by most of the elderly residing in the community. Cohabiting with children and relying on care from family was still the primary desired aging care model for seniors (78 %), followed by living in institutions (14.8 %) and living at home independently while relying on community resources (7.2 %). Education, monthly income, number of family and friend helpers, current living situation, and distance to healthcare centers had statistically significant effects on preferences for care in facilities or co-residing with children.

The results of this study extend the Andersen-Newman Behavioural Model in two ways. On the one hand, findings suggested the significant influence of informal care in aging care arrangement preferences, which was originally neglected in the Andersen conceptual framework [28]. Interestingly, this study found that elders having less social support were more likely to rely on communities compared with living at home and living in institutions. Shuval’s study in Israel indicated that isolated individuals with few social supports often seek services as s substitute for new ties [35]. In China, while home care is informal and institutional care is formal, community care was a mix of formal and informal care. When receiving care in the community care, elderly people have great potential to develop new social ties, which may explain why living in the community was preferred to home care and institutional care among elderly with fewer social supports. William’s study on elderly care in the United States also suggested that despite a desire to limit the extension of aging care institutions, there are great advantages to mixing formal and informal care in homes, communities and institutions, which was a major policy issue to focus in the future [34].

On the other hand, findings suggested the importance of availability to community care services in elderly care model choices. This finding confirmed the influence of organizational factors (e.g. the structure of a national health system) as an enabling resource. Our study found that short distances to CHC reduced the probability of preferring care from family, compared with preferring to rely on community resources, which was consistent with previous studies. For example, Zhang Wenjuan’s result indicated that having a healthcare center within the community lowered the intention for institutionalization by 26.9 % among elderly without functional disabilities [10]. Functional disability refers to any impairment in the patient’s ability to perform essential activities of everyday life, including feeding, bathing, dressing, toileting, grooming and physical ambulation, according to ADL. Greene’s study also found that personal-care aides and housekeeping services lowered the probability of institutionalization for those with severe functional disabilities [36]. Bilsen’s study in the Netherlands indicated that community-based social services for elders could delay their admittance to institutionalized settings [18]. While in our study, community based social service organizations (including home based care centers, day care centers, and activity centers) did not have statistically effects on the choice of desired aging care arrangements for those still living outside of institutions. This difference may result from differences in the population that was sampled, the different models used in their studies, or the low level of prevalence and availability of the community based social services and organizations (see Table 2) in the sampling districts.

CHCs play an important role in the community life of elderly. CHCs were felt to be necessary by almost all (94.6 %) of those surveyed and were located within 2 km of most (89.1 %) of the elderly surveyed. However, according to existing studies, CHC service satisfaction was not very high. For example, Yang Jun’s study, which covered 52 cities nationwide, showed that more than half of doctors and nurses employed in community health centers had low-level medical training (i.e. fewer than 3 years post–high or 4 years post-middle school medical training) [37], and community residents showed marked distrust of the doctors, nurses and service quality. But people tend to be satisfied with its fast access and affordability to treatments and medicines [17, 37].

Policy makers should consider improving CHC’s service quality, strengthening the medical or professional training of doctors and nurses, enriching its service package by adding services such as home-care service and day-care service in order to relieve the burden on family and institutions.

It was interesting that our results indicated that need variables (including ADL, IADL and chronic diseases) was not associated with elders’ preference for aging care models, in contrast to predisposing variables (education), enabling variables (monthly income, number of family and friend helpers and current living arrangements) and community variables (distance to CHCs). It supports Shu’s opinion that in China, aging care preferences are the result of balancing family care resources, economic resources and culture norms while in western countries, health or disability condition plays a stronger role in the aging care arrangement preference [5]. A recent study on elder homes in Tianjin city found that the majority of their residents had no need for assistance in daily life activities, and the major reasons for their institutionalization was the unavailability of children, including being childless [38].

This study was the first to use multi-category options to present the aging care models and examine the associated factors among Chinese community elderly residents living outside of institutions. However, this study had some limitations worth mentioning. First, it only examined the effect of community-based facility and service availability, not utilization, which should be studied in the near future. Second, this study sampled elderly people only from urban and suburb areas, and could not represent the situation in rural areas in China. Third, social values and norms of filial piety were not included in the model, which should be an important predisposing factor affecting the aging care preference. Fourth, given three options of the future aging care models, some elderly likely want a combination of some of these features, for example, living with children but having an outside nurse come in to help with bathe; or living independently but relying on children for transportation and grocery shopping. Although we framed the questions and asked the elderly to choose the most desired aging care model, forced choices may still result in the neglect of the possibility of some combining aging care arrangements. Fifth, there was a bias in only selecting people who live in households, and excluding those already living in institutions. People usually preferred what they already had; therefore excluding the institutionalized elderly may result in the under-representation of elderly who preferred living in institutions in the future. However, despite these limitations, it was still meaningful to interpret the perspectives of elderly who live in the community. The results of this analysis provide new insight into the current community capacity in supporting senior citizens and its effect on elderly aging care preference.

## Conclusions

In Beijing, China, aging care preferences are the result of balancing family care resources, economic resources and social status, and the accessibility of community resources. Cohabiting with children and relying on family care was still the most desired care model for Chinese community-dwelling senior citizens. Community facilities and services supporting the elderly were found to be insufficient in our sample from Beijing. Only a small percent of elderly considered relying on community support for their remaining life. Enriching community capacities to support the elderly and encouraging the elderly to utilize community services could reduce the pressure on families and institutions to provide elderly care. For China’s future health system, efforts should be made to improve community capacity to provide integrated services to senior citizens.

## Additional file

Additional file 1: Sampling progress. (DOCX 96 kb)

## Abbreviations

ADL: Activity of Daily Living
CHC: Community health centers
IADL: Instrumental Activities of Daily Living
