# Supplementary material for: Identifying community healthcare supports for the elderly and the factors affecting their aging care model preference: evidence from three districts of Beijing
Source: BMC Health Serv Res. 2016 Nov 15;16(Suppl 7):83–92. doi: 10.1186/s12913-016-1863-y (PMC5123297; doi:10.1186/s12913-016-1863-y)
Supplement: Additional file 1: — Sampling progress. (DOCX 96 kb) [file 12913_2016_1863_MOESM1_ESM.docx]

# Identifying community healthcare supports for the elderly and the factors affecting their aging care model preference: Evidence from three districts of Beijing

Tianyang Liu^1^, XiaoningHao^1§^, Zhenzhong Zhang^1^

# Additional files

### Additional file 1. Sampling progress

**Step 1:**

The sample size of this survey is defined using formula N=Z2× (P × (1-P))/E2, where N is the sample size, Z2 is the abscissa of the normal curve that cuts off an area α at the tails (1 - α equals the desired confidence level), E is the desired level of precision; p is the estimated proportion of an attribute that is present in the population. Here Z=1.96, E=3%, P=0.5, and N=1067. The initial sample size is 1067.

**Step 2:**

This study used the survey “Health and Social Support of Elderly Population in Community”. The questionnaires were issued between January and May 2013, covering three different functional districts (Xicheng, Chaoyang and Tongzhou) of Beijing. Beijing is divided into four different functional districts: Core area of political and culture function (including Dongcheng, Xicheng, Chongwen, Xuanwu and Shijingshan districts); district of extended urban functions (Chaoyang, Haidian and Fengtai districts); district of new development (Tongzhou, Daxing, Shunyi, and Changping); and district of ecology (including Mentougou, Fangshan, Pinggu, Huairou, Miyun and Yanqing districts). This survey chooses three districts of different functions (except for the ecology function) to represent the situation of Beijing and other first-tier cities like Shanghai and Guangzhou. See additional table 1.

**Additional table 1. Districts sampled**

| **District name** | **District type** | **District function** |
| --- | --- | --- |
| Xicheng | Urban | Core area of political and culture functions |
| Chaoyang | Urban | District of extended urban functions |
| Tongzhou | Suburb | District of new development |

**Step 3:**

The 1067 sample size then was distributed by districts, age and gender. See table 2 and table **Additional table 2. Sample size and questionnaire completion by district**

| **Districts** | **Sample size of elders distributed by districts and the sample size of communities** | | | | | **Questionnaires finished** | |
| --- | --- | --- | --- | --- | --- | --- | --- |
|  | **Population of 60 years or more（10,000）** | **percentage** | **Sample size distributed by percentage** | **Adjusted sample size** | **Sample of communities (villages)** | **Number of questionnaires finished** | **percentage** |
| Xicheng | 30.3 | 33.04% | 354 | 354 | 14 | 350 | 32.32% |
| Chaoyang | 42.8 | 46.67% | 499 | 499 | 20 | 506 | 46.72% |
| Tongzhou | 18.6 | 20.28% | 217 | 217 | 9 | 227 | 20.96% |
| Total | 91.7 | 100.00% | 1067 | 1067 | 43 | 1083 | 100.00% |

| **Sample size of elders distributed by districts by age and gender** | | | | | | | **Questionnaires finished** | | | |  |
| --- | --- | --- | --- | --- | --- | --- | --- | --- | --- | --- | --- |
|  | **Age** | **Population of 60 years or more in Beijing**  **（10,000）** | **percentage** | **male** | **female** | **Sample size distributed by percentage** | **Number of questionnaires finished** | **percentage** | **Male** | **female** | **Questionnaires distributed by percentage** |
|  |  |  |  | **（10,000）** | **（10,000）** |  |  |  |  |  |  |
| 1 | 60-69 | 118.7 | 47.90% | 119  (48%) | 128.9  （52%） | 900 | 476 | 43.95% | 540  （49%） | 543  （51%） | 905 |
| 2 | 70-79 | 90.6 | 36.60% |  |  |  | 429 | 39.61% |  |  |  |
| 3 | 80-89 | 36 | 14.50% |  |  | 170 | 163 | 15.05% |  |  | 178 |
| 4 | 90+ | 2.6 | 1.00% |  |  |  | 15 | 1.39% |  |  |  |
| Total | | 247.9 | 100.00% | 247.9 | | 1070 | 1083 | 100.00% | 1083 | | 1083 |

**Additional table 3. Sample size and questionnaire completion by age**

**Step 4:**

(1) 29 interviewers were recruited and trained for the door to door interview (face to face). Interviews were conducted within the 43 randomly selected communities (villages), and in each community, 24 to 26 elders will be found for interview. In the selected community, the distribution map of all of the households was drawn and the number of households in each building will be counted. Doors were knocked by systematic sampling method (For example, door number 101, 105, 110, 115…). And the interviewers will stop to knock doors when 24 to 26 elders were interviewed in one community.

(2) Elders who were aged more than 60, living in the community (village) for more than 6 months, having Beijing citizenship, and having the mental capability for the interview were eligible to participate in the study. And if there were several elders in one household, only one elder was eligible.
